# Lifestyle correlates of eight breast cancer-related metabolites: a cross-sectional study within the EPIC cohort

**DOI:** 10.1186/s12916-021-02183-2

**Published:** 2021-12-10

**Authors:** Mathilde His, Vivian Viallon, Laure Dossus, Julie A. Schmidt, Ruth C. Travis, Marc J. Gunter, Kim Overvad, Cecilie Kyrø, Anne Tjønneland, Lucie Lécuyer, Joseph A. Rothwell, Gianluca Severi, Theron Johnson, Verena Katzke, Matthias B. Schulze, Giovanna Masala, Sabina Sieri, Salvatore Panico, Rosario Tumino, Alessandra Macciotta, Jolanda M. A. Boer, Evelyn M. Monninkhof, Karina Standahl Olsen, Therese H. Nøst, Torkjel M. Sandanger, Antonio Agudo, Maria-Jose Sánchez, Pilar Amiano, Sandra M. Colorado-Yohar, Eva Ardanaz, Linda Vidman, Anna Winkvist, Alicia K. Heath, Elisabete Weiderpass, Inge Huybrechts, Sabina Rinaldi

**Affiliations:** 1 International Agency for Research on Cancer (IARC/WHO), Nutrition and Metabolism Branch, 150 cours Albert Thomas, 69372, CEDEX 08 Lyon, France; 2 Cancer Epidemiology Unit, Nuffield Department of Population Health, University of Oxford, Oxford, UK; 3 Department of Public Health, Aarhus University, Aarhus, Denmark; 4 Danish Cancer Society Research Center, Copenhagen, Denmark; 5 Department of Public Health, Section of Environmental Health, Faculty of Health and Medical Sciences, University of Copenhagen, Copenhagen, Denmark; 6 Université Paris-Saclay, UVSQ, Inserm, CESP U1018, “Exposome and Heredity” team, Gustave Roussy, Villejuif, France; 7 Department of Statistics, Computer Science, Applications “G. Parenti”, University of Florence, Florence, Italy; 8 Department of Cancer Epidemiology, German Cancer Research Center (DKFZ), Heidelberg, Germany; 9 Department of Molecular Epidemiology, German Institute of Human Nutrition, Nuthetal, Germany; 10 Institute of Nutritional Science, University of Potsdam, Potsdam, Germany; 11 Institute for Cancer Research, Prevention and Clinical Network (ISPRO), Florence, Italy; 12 Epidemiology and Prevention Unit, Fondazione IRCCS Instituto Nazionale dei Tumori di Milano, Milan, Italy; 13 Dipartimento Di Medicina Clinica E Chirurgia, Federico Ii University, Naples, Italy; 14 Cancer Registry and Histopathology Department, Provincial Health Authority (ASP 7) Ragusa, Ragusa, Italy; 15 Department of Clinical and Biological Sciences, University of Turin, Turin, Italy; 16 Center for Nutrition, Prevention, and Health Services, National Institute for Public Health and the Environment (RIVM), Bilthoven, 3720 BA the Netherlands; 17 Julius Center for Health Sciences and Primary Care, University Medical Center Utrecht, Utrecht University, Utrecht, the Netherlands; 18 Department of Community Medicine, Faculty of Health Sciences, UiT The Arctic University of Norway, NO-9037 Tromsø, Norway; 19 Unit of Nutrition and Cancer, Catalan Institute of Oncology - ICO, L’Hospitalet de Llobregat, Spain; 20 Nutrition and Cancer Group; Epidemiology, Public Health, Cancer Prevention and Palliative Care Program; Bellvitge Biomedical Research Institute - IDIBELL, L’Hospitalet de Llobregat, Spain; 21 Escuela Andaluza de Salud Pública (EASP), Granada, Spain; 22 Instituto de Investigación Biosanitaria ibs.GRANADA, Granada, Spain; 23 Centro de Investigación Biomédica en Red de Epidemiología y Salud Pública (CIBERESP), Madrid, Spain; 24 Department of Preventive Medicine and Public Health, University of Granada, Granada, Spain; 25 Ministry of Health of the Basque Government, Sub-Directorate for Public Health and Addictions of Gipuzkoa, San Sebastián, Spain; 26 Biodonostia Health Research Institute, Group of Epidemiology of Chronic and Communicable Diseases, San Sebastián, Spain; 27 CIBER Epidemiology and Public Health (CIBERESP), Instituto de Salud Carlos III (ISCIII), Madrid, Spain; 28 Department of Epidemiology, Murcia Regional Health Council, IMIB-Arrixaca, Murcia, Spain; 29 Research Group on Demography and Health, National Faculty of Public Health, University of Antioquia, Medellín, Colombia; 30 Navarra Public Health Institute, Pamplona, Spain; 31 IdiSNA, Navarra Institute for Health Research, Pamplona, Spain; 32 Department of Radiation Sciences, Oncology, Umeå University, Umeå, Sweden; 33 Sustainable Health, Department of Public Health and Clinical Medicine, Umeå University, Umeå, Sweden; 34 Department of Epidemiology and Biostatistics, School of Public Health, Imperial College London, London, UK; 35 International Agency for Research on Cancer (IARC/WHO), Office of the Director, Lyon, France

**Keywords:** Metabolites, Breast cancer, Cross-sectional, Lifestyle, Anthropometry

## Abstract

**Background:**

Metabolomics is a promising molecular tool for identifying novel etiological pathways leading to cancer. In an earlier prospective study among pre- and postmenopausal women not using exogenous hormones, we observed a higher risk of breast cancer associated with higher blood concentrations of one metabolite (acetylcarnitine) and a lower risk associated with higher blood concentrations of seven others (arginine, asparagine, phosphatidylcholines (PCs) aa C36:3, ae C34:2, ae C36:2, ae C36:3, and ae C38:2).

**Methods:**

To identify determinants of these breast cancer-related metabolites, we conducted a cross-sectional analysis to identify their lifestyle and anthropometric correlates in 2358 women, who were previously included as controls in case-control studies nested within the European Prospective Investigation into Cancer and Nutrition cohort and not using exogenous hormones at blood collection. Associations of each metabolite concentration with 42 variables were assessed using linear regression models in a discovery set of 1572 participants. Significant associations were evaluated in a validation set (*n* = 786).

**Results:**

For the metabolites previously associated with a lower risk of breast cancer, concentrations of PCs ae C34:2, C36:2, C36:3, and C38:2 were negatively associated with adiposity and positively associated with total and saturated fat intakes. PC ae C36:2 was also negatively associated with alcohol consumption and positively associated with two scores reflecting adherence to a healthy lifestyle. Asparagine concentration was negatively associated with adiposity. Arginine and PC aa C36:3 concentrations were not associated to any of the factors examined. For the metabolite previously associated with a higher risk of breast cancer, acetylcarnitine, a positive association with age was observed.

**Conclusions:**

These associations may indicate possible mechanisms underlying associations between lifestyle and anthropometric factors, and risk of breast cancer. Further research is needed to identify potential non-lifestyle correlates of the metabolites investigated.

**Supplementary Information:**

The online version contains supplementary material available at 10.1186/s12916-021-02183-2.

## Background 

Metabolomics is an important tool in the identification of new etiological pathways associated with chronic diseases, including breast cancer [1–8], as the metabolome reflects both endogenous parameters and exogenous exposures [9]. Prospective studies using targeted metabolomics (analyses of a pre-defined panel of metabolites) or untargeted metabolomics approaches have reported novel associations of pre-diagnostic blood concentrations of endogenous metabolites with breast cancer risk. These metabolites include lysophosphatidylcholine a C18:0 [8], 16a-hydroxy-DHEA-3-sulfate [4, 5], various carnitines [4, 5], caprate (10:0) [6], histidine, glycerol, N-acetyl-glycoprotein [7], acetone, glycerol-derived compounds, other amino acids, and lipids [2, 3], suggesting new potential avenues of research and possible additional targets for prevention.

In a previous case-control study nested within the European Prospective Investigation into Cancer and Nutrition (EPIC) cohort, we investigated the association between blood concentrations of endogenous metabolites, measured by targeted metabolomics, and risk of breast cancer [1]. We reported a positive association between acetylcarnitine (C2) and breast cancer risk and negative associations of arginine, asparagine, phosphatidylcholines acyl-alkyl (PCs ae) C36:3, C34:2, C36:2, C38:2, and phosphatidylcholine diacyl (PC aa) C36:3 with breast cancer risk, among women not using exogenous hormones at blood collection.

To further assess how these findings can inform breast cancer prevention research, a better understanding of potentially modifiable determinants of blood levels of these metabolites is needed. Towards this aim, we report here the results of a cross-sectional analysis nested in the EPIC cohort to investigate associations of a wide range of lifestyle and anthropometric variables and acetylcarnitine, arginine, asparagine, PCs aa C36:3, ae C34:2, ae C36:2, ae C36:3, and ae C38:2.

## Methods

### The EPIC study

EPIC is an ongoing multi-center cohort study including approximately 520,000 participants recruited between 1992 and 2000 from ten European countries [10]. Female participants (*n* = 367,903) were aged 35–75 years at recruitment. Detailed information was collected on dietary, lifestyle, reproductive, medical, and anthropometric data at inclusion [10]. Around 246,000 women from all countries provided a baseline blood sample. Blood was collected according to a standardized protocol in France, Germany, Greece, Italy, the Netherlands, Norway, Spain, and the UK [10]. Serum (except in Norway), plasma, erythrocytes, and buffy coat aliquots were stored in liquid nitrogen (−196°C) in a centralized biobank at IARC. In Denmark, blood fractions were stored locally in the vapor phase of liquid nitrogen containers (−150°C), and in Sweden, they were stored locally at −80°C in standard freezers. All participants provided written informed consent to participate in the EPIC study. This study was approved by the ethics committee of the International Agency for Research on Cancer (IARC) and all centers.

### Study population and cross-sectional design

This study included all female EPIC participants (1) who provided a blood sample; (2) who were previously included in one of six case-control studies on cancer etiology nested within the EPIC cohort (on breast [1], endometrial [11], colorectal [12], kidney [13], liver [14], and gallbladder cancers) with available blood concentrations of acetylcarnitine, arginine, asparagine, PCs aa C36:3, ae C34:2, ae C36:2, ae C36:3, and ae C38:2 measured by the same targeted metabolomics approach; (3) who were included as control participants in these studies (i.e., free of cancer (except non-melanoma skin cancer) at the time of the diagnosis of the cases, using incidence-density sampling, and matched to cases by age, sex, study center, time of blood collection, fasting status at blood collection (except for kidney cancer study), menopausal status and exogenous hormone use at blood collection (for breast, endometrial, liver, and gallbladder studies), and phase of menstrual cycle (for breast and endometrial cancer studies)); and (4) whose samples were included in an analytical batch including at least 10 samples, to ensure proper normalization of metabolite concentrations (see the “Statistical analyses” section) (*N* = 3163).

We then excluded women who declared use of hormones at blood collection (*n* = 768), and those whose hormone use status at blood collection was unknown (*n* = 37), because associations between the studied metabolites and breast cancer risk were limited to hormone non-users [1]. The current analysis included data from 2358 participants.

The 2358 participants were split into a discovery set (*N* = 1572, 66.7% of the population) and a validation set (*N* = 786, 33.3% of the population). Metabolites of interest were those found to be associated with breast cancer risk, and this observed association could result from associations between metabolites and some of the correlates under study in the present work. Thus, the discovery set included all controls from the breast cancer study (*n* = 1079), and randomly selected controls from the other nested case-control studies (*n* = 493), while the validation set did not include participants from the breast cancer study. This way, associations identified on the discovery set and further validated on the validation set are guaranteed not to be driven by the breast cancer study only.

### Laboratory measurements

Before exclusions of hormone users, a total of 3179 samples were available for 3163 women. All samples, plasma (in 95.1% of samples) or serum, were assayed by liquid chromatography-mass spectrometry using the AbsoluteIDQ p180 commercial kit (Biocrates Life Sciences AG, Innsbruck, Austria). A total of 2289 (72.0%) samples were assayed at the laboratory of the Biomarkers Group at IARC (breast, colorectal, kidney, and liver cancer studies); 851 (26.8%) at the Imperial College, London; and 39 (1.2%) at the Helmholtz Zentrum, München, Germany. At IARC, analyses were run on a QTRAP5500 (breast, kidney, and liver cancer studies) and TQ4500 (colorectal cancer study) mass spectrometers (AB Sciex, Framingham, MA, USA), while at the Imperial College London and Helmholtz Zentrum, analyses were run using an API4000TQ (endometrial and gallbladder cancer studies). All analyses for a given study were performed using the same instrument. Sixteen participants had their samples analyzed in two different studies, at IARC and at the Helmholtz Zentrum, for whom the metabolite concentrations were averaged over the two measures.

Out of the 3179 samples, arginine concentrations could not be quantified in five, as they were below the lower limit of quantification (LLOQ) and were therefore imputed to half this LLOQ, consistently with previous work [1].

### Covariate data

Details of data collection in EPIC are described elsewhere [10]. Lifestyle and medical factors were assessed in the baseline questionnaire. Usual dietary intakes were assessed using center- or country-specific validated questionnaires covering the previous 12 months and matched to the US Department of Agriculture food composition database to estimate macronutrient intakes [15]. Glycemic index and glycemic load were computed. In all EPIC centers, except France, Oxford, and Norway, height, weight, and waist and hip circumference were measured on all participants using similar protocols (in Umeå (Sweden), only weight and height were measured). In France and Oxford, weight, height, and waist and hip circumferences were measured in a sub-set of participants, but self-reported weight and height were obtained from all individuals, and validation studies showed high correlations between self-reported and measured values (*r* ≥ 0.90) [16, 17]. In Oxford, self-reported measurements also included waist and hip circumferences. In Norway, only self-reported height and weight were available.

Dietary data were used to compute the inflammatory score of the diet (ISD) [18] (reflecting the inflammatory potential of the diet based on 28 dietary components), the modified Mediterranean diet score [19] (a 9-component score indicating the degree of adherence to the traditional Mediterranean diet; 0 minimal adherence to 9 maximal adherence), and the Diet Quality Index-International (DQI-I; a 17-component score based on general nutritional guidelines [20, 21]; 0 to 100, minimal to maximal diet quality). Dietary and lifestyle data were combined to calculate the Healthy Lifestyle Index (HLI) [22], designed to reflect five components of lifestyle factors (smoking, alcohol consumption, diet (cereal fibers, red and processed meat, the ratio of polyunsaturated to saturated fatty acids, margarine, glycemic load, and fruits and vegetables), physical activity, and body mass index; ranging from 0, least healthy, to 20). Furthermore, we calculated the World Cancer Research Fund/American Institute for Cancer Research score, which reflects recommendations for cancer prevention on weight maintenance, physical activity, intake of food and drinks which promote weight gain, of plant-based foods, of animal-based foods, of alcohol, and breastfeeding [23] (from 0, low adherence to recommendation, to 7 for women).

### Statistical analyses

#### Normalization of metabolite concentrations

A specific statistical pipeline was developed [24] and applied on raw metabolite concentrations (before exclusion of hormone users) to adequately pool measures obtained from different studies, instruments, and laboratories. This pipeline was shown to be efficient in removing unwanted variability and improving the comparability of measurements acquired across different nested studies. Log-transformed concentrations of the metabolites of interest were normalized to remove effects of analytical batch and study, which were estimated as random effects in mixed-effects linear models correcting for possible heteroscedasticity. Corrected metabolite concentrations analyzed in this work correspond to residuals from the model.

#### Missing data

When missing values on covariates represented less than 5% of the overall values, they were imputed to the mode value (categorical variables: number of full-term pregnancies, ever use of oral contraceptive, ever use of hormones for menopause (by menopausal status), education level, physical activity, smoking status, fasting status) or median (continuous variables: age at menarche, age at first full-term pregnancy (among parous women), duration of breastfeeding among women who breastfed, waist circumference, hip circumference, waist/hip ratio, time at blood collection). When missing values represented more than 5% of values for a variable, this variable was categorized, and a “missing” category was created (phase of menstrual cycle at blood collection for pre- and perimenopausal women, breastfeeding, lifetime alcohol consumption, Healthy Lifestyle Index, WCRF/AICR score).

#### Identification of correlates

Participants’ characteristics were described using frequencies for categorical variables and mean (standard deviation) for continuous variables. We calculated partial Pearson’s correlations between metabolite concentrations (adjusted for center and age) and between metabolites and age (adjusted for center).

Analyses were first run in the discovery set. For each metabolite of interest and each lifestyle variable, a linear regression model was built with metabolite concentration as a dependent variable. Models were adjusted for center of recruitment, age at blood collection, menopausal status (premenopausal, perimenopausal, postmenopausal [25]), phase of the menstrual cycle for premenopausal women (follicular, ovulatory, luteal, missing), time of the day, and fasting status at blood collection (“No”: < 3 h since last meal (< 4 h in Umeå), “In between”: 3–6 h (4–8 h in Umeå), and “Yes”: > 6 h (> 8 h in Umeå)). Models that examined age as exposure were not adjusted for age, and models with menopausal status as main exposure were not adjusted for phase of menstrual cycle, as this variable is defined in premenopausal women only.

Variables tested as possible correlates were age at blood collection (continuous), age at menarche (continuous), total duration of menstrual cycles (quartiles/missing), pregnancy (ever/never), number of full-term pregnancies (continuous), age at first full-term pregnancy (nulliparous/quartiles), breastfeeding (ever/never/missing), duration of breastfeeding (nulliparous/quartiles/missing), use of oral contraceptive (ever/never; current users excluded), menopausal status at blood collection (premenopausal/perimenopausal/postmenopausal), use of hormones for menopause (ever/never; current users are excluded), education level (no schooling or primary/technical, professional or secondary/longer education), physical activity (Cambridge Index [26]: inactive/moderately inactive/moderately active/active), smoking status (never/former/current), smoking status combined with intensity (never/current, 1–15 cigarettes/day/current, 16+ cigarettes/day/current, pipe/cigar/occasional/former, quit for ≤10 years/former, quit 11–20 years/former, quit > 20 years), baseline alcohol consumption (continuous, g/day), lifetime alcohol consumption (non-drinker/former drinker/current > 0–3 g/day/> 3–12 g/day/> 12–24 g/day/> 24 g/day/missing), BMI (continuous, kg/m^2^), waist circumference (continuous, cm), hip circumference (continuous, cm), waist/hip ratio (continuous), height (continuous, cm), total energy intake (continuous, kcal/day), and the following food components estimated as residuals on total energy intake (continuous, g/day): protein, carbohydrate, starch, sugar, fiber, fat (total), fatty acids (monounsaturated, polyunsaturated, saturated, trans, trans-monoenoic, trans-polyenoic), glycemic index (continuous), glycemic load (continuous), Healthy Lifestyle Index (0–10/11–15/16–20), WCRF/AICR score (quartiles/missing), modified Mediterranean diet score (continuous), diet quality index (continuous), and inflammatory score of the diet (continuous).

For each metabolite, *P*-values from *F*-tests for each variable were collected and were corrected for multiple testing by controlling for family-wise error rate at *α* = 0.05 by permutation-based stepdown *minP* adjustment of *P*-values, a method which accounts for dependencies between tests [27].

#### Validation

All statistically significant associations in the discovery set (based on *P*-values corrected for multiple tests ≤0.05) were assessed in the validation set, using the same model and categories of variables as in the discovery set. In this validation set, a more conservative approach was chosen for controlling for multiple tests [28], i.e., the Bonferroni correction based on the number of tests run for each metabolite.

For all variables showing a significant association with the metabolites of interest in both the discovery and validation sets, continuous variables were categorized (quartiles) and means of metabolites, with 95% confidence intervals, were estimated in each category, using the overall dataset (*n* = 2358).

#### Interactions

For each metabolite and each variable examined as potential correlate, we investigated interaction with fasting status (no/in between/yes), menopausal status at blood collection (pre-/peri-/postmenopausal), and BMI (18.5–24.9/25–29.9/≥30 kg/m^2^, excluding *n* = 15 participants with BMI < 18.5 kg/m^2^), in the discovery set. To do so, an interaction term was added in the model and the *P*-value associated with this term was evaluated, after correction for multiple testing using the permutation *minP* algorithm.

#### Sensitivity analyses

We conducted sensitivity analyses (1) excluding participants from the liver and gallbladder studies (*n* = 128), for which the blood fraction analyzed was serum and not plasma, and (2) excluding participants with self-reported diabetes (*n* = 71) or with missing data on diabetes status (*n* = 160) at recruitment.

## Results

Participants’ characteristics overall and from the discovery and validation sets are shown in Table 1. Overall, 39.7% of the participants were not fasting at blood collection while 44.4% were considered fasting (more than 6 h since last meal (8 h in Umeå)). Around 30% of participants were premenopausal. Overall, participant characteristics were similar among discovery and validation sets (Table 1). Of note, the mean age (standard deviation (*SD*)) at blood collection in the validation set was 55.5 (8.1) years and 53.1 (8.6) years in the discovery set. Consequently, the proportion of postmenopausal women was 61.8% in the validation set and 51.4% in the discovery set. In the validation set, 42.0% of participants had ever used oral contraceptive (vs 50.3% in the discovery set), 53.3% of women had received none or primary education (vs 47.3% in discovery set), 29.9% were physically inactive (vs 24.7% in discovery set), 16.9% were current smokers (vs 21.6% in discovery set), and 26.3% were alcohol non-consumers (vs 19.2% in discovery set).

**Table 1:** Main characteristics of women included (hormone non-users only), overall and in discovery and validation sets

	Overall (***n*** = 2358)	Discovery (***n*** = 1572)	Validation (***n*** = 786)
Age at blood collection (years)	53.9 (8.5)	53.1 (8.6)	55.5 (8.1)
Fasting status at blood collection^a^ (%)			
No	936 (39.7)	639 (40.6)	297 (37.8)
In between	375 (15.9)	252 (16.0)	123 (15.6)
Yes	1047 (44.4)	681 (43.3)	366 (46.6)
Menopausal status at blood collection (%)			
Premenopausal	722 (30.6)	522 (33.2)	200 (25.4)
Postmenopausal	1294 (54.9)	808 (51.4)	486 (61.8)
Perimenopausal	342 (14.5)	242 (15.4)	100 (12.7)
Age at first menstrual periods (years) (mean (*SD*))	13.1 (1.6)	13.1 (1.6)	13.1 (1.6)
Number of full-term pregnancies (mean (*SD*))	2.1 (1.3)	2.0 (1.2)	2.1 (1.4)
Age at first full-term pregnancy (years) (mean (*SD*))	25.2 (4.3)	25.1 (4.4)	25.4 (4.2)
Breastfeeding (in parous women) (%)			
Yes	1669 (80.9)	1110 (80.7)	559 (81.2)
No	280 (13.6)	181 (13.2)	99 (14.4)
Missing	115 (5.6)	85 (6.2)	30 (4.4)
Ever used oral contraceptive (%)	1120 (47.5)	790 (50.3)	330 (42.0)
Ever used MHT (%)	297 (12.6)	198 (12.6)	99 (12.6)
Education level (%)			
Primary/no schooling	1162 (49.3)	743 (47.3)	419 (53.3)
Technical/professional/secondary	819 (34.7)	560 (35.6)	259 (33.0)
Longer education	377 (16.0)	269 (17.1)	108 (13.7)
Physical activity (Cambridge Index) (%)			
Inactive	623 (26.4)	388 (24.7)	235 (29.9)
Moderately inactive	929 (39.4)	623 (39.6)	306 (38.9)
Moderately active	450 (19.1)	307 (19.5)	143 (18.2)
Active	356 (15.1)	254 (16.2)	102 (13.0)
Smoking status (%)			
Never	1406 (59.6)	934 (59.4)	472 (60.1)
Former	480 (20.4)	299 (19.0)	181 (23.0)
Smoker	472 (20.0)	339 (21.6)	133 (16.9)
Alcohol consumption at recruitment (%)			
Non-drinker	509 (21.6)	302 (19.2)	207 (26.3)
> 0–3 g/day	707 (30.0)	482 (30.7)	225 (28.6)
> 3–12 g/day	619 (26.3)	416 (26.5)	203 (25.8)
> 12–24 g/day	337 (14.3)	239 (15.2)	98 (12.5)
> 24 g/day	186 (7.9)	133 (8.5)	53 (6.7)
Height (cm) (mean (*SD*))	160.4 (6.8)	160.6 (6.7)	160.0 (6.8)
BMI (kg/m^2^) (mean (*SD*))	26.0 (4.3)	25.9 (4.3)	26.3 (4.4)
Waist circumference (cm) (mean (*SD*))	81.9 (10.6)	81.5 (10.4)	82.6 (10.9)
Waist/hip ratio (mean (*SD*))	0.80 (0.07)	0.80 (0.07)	0.81 (0.07)
Total energy intake (kcal/day) (mean (*SD*))	2010.3 (547.7)	2016.3 (557.6)	1998.3 (527.4)
Healthy Lifestyle Index^b^ (mean(*SD*))	12.6 (3.0)	12.6 (3.0)	12.8 (2.9)
WCRF/AICR score^c^ (mean (*SD*))	3.9 (1.0)	3.9 (1.0)	3.9 (1.0)
Modified Mediterranean diet score (mean (*SD*))	4.3 (1.8)	4.3 (1.8)	4.5 (1.7)
Inflammatory score of the diet (mean (*SD*))	0.9 (1.7)	0.89 (1.7)	0.8 (1.7)
Diet Quality Index-International (mean (*SD*))	57.4 (7.8)	57.2 (7.9)	57.8 (7.7)
**Metabolite concentrations (normalized), μmol/L**			
Arginine (geometric mean (*SD*))	64.1 (2.6)	64.2 (2.6)	63.9 (2.6)
Asparagine (geometric mean (*SD*))	41.5 (2.7)	41.4 (2.7)	41.6 (2.7)
C2 (geometric mean (*SD*))	4.9 (2.7)	4.9 (2.7)	4.9 (2.6)
PC aa C36:3 (geometric mean (*SD*))	130.0 (2.6)	127.0 (2.6)	136.0 (2.7)
PC ae C34:2 (geometric mean (*SD*))	12.2 (2.7)	12.1 (2.8)	12.4 (2.7)
PC ae C36:2 (geometric mean (*SD*))	15.6 (2.7)	15.5 (2.7)	15.6 (2.7)
PC ae C36:3 (geometric mean (*SD*))	8.1 (2.7)	8.0 (2.7)	8.2 (2.7)
PC ae C38:2 (geometric mean (*SD*))	2.1 (2.7)	2.1 (2.7)	2.1 (2.8)

*Abbreviations*: *AICR* American Institute for Cancer Research, *BMI* body mass index, *C2* acetylcarnitine, *MHT* menopause hormone therapy, *PC aa* phosphatidylcholine diacyl, *PC ae* phosphatidylcholine acyl-alkyl, *SD* standard deviation, *WCRF* World Cancer Research Fund

^a^No: < 3 h since last meal (< 4 h in Umeå); in between: 3–6 h since last meal (4–8 h in Umeå); yes: > 6 h since last meal (> 8 h in Umeå)

^b^Healthy Lifestyle Index was missing for 144 (6.1%) participants

^c^WCRF/AICR score was missing for 196 (8.3%) participants

**Fig. 1:**
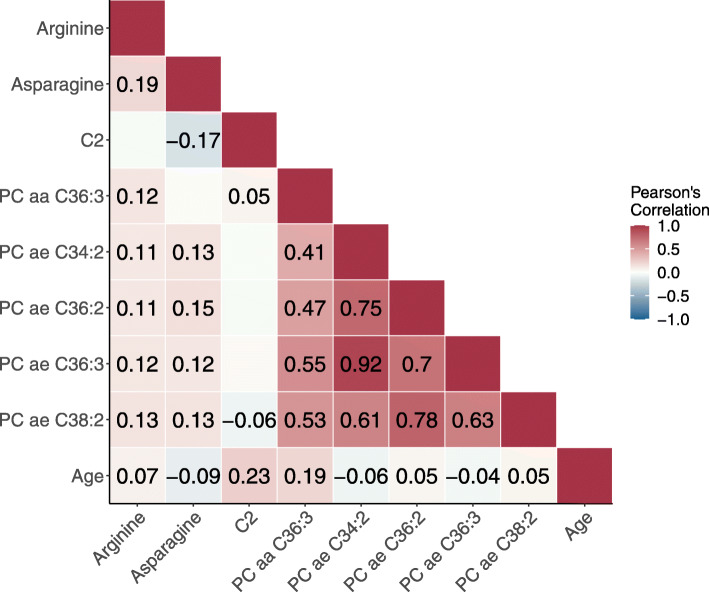
Partial Pearson correlations between metabolites identified as associated with breast cancer risk, and age (*N* = 2358). Metabolite concentrations were log-transformed and normalized as described in the “Methods” section. Coefficients are shown only for significant correlations (*P*-value < 0.05). Correlations between metabolite concentrations are adjusted for center and age, and correlations between metabolites and age are adjusted for center. Abbreviations: C2, acetylcarnitine; PC aa, phosphatidylcholine diacyl; PC ae, phosphatidylcholine acyl-alkyl

In all participants (*N* = 2358), strong correlations were observed between acyl-alkyl PCs (Fig. 1, Pearson’s correlation coefficients 0.61 to 0.92), while moderate correlations were observed between acyl-alkyl PCs and PC aa C36:3 (0.41 to 0.55). Arginine was moderately correlated with all metabolites except for acetylcarnitine (C2), with an observed correlation of 0.19 with asparagine and correlations ranging from 0.11 to 0.13 with PCs. Asparagine showed similar low correlations (0.12 to 0.15) with PCs and a negative correlation with C2 (−0.17). C2 showed the greatest correlation with age (0.23), followed by PC aa C36:3 (0.19), while for other metabolites correlations with age ranged from −0.09 to 0.07.

In the discovery set, 104 associations (31% of the 336 associations tested, 8 metabolites × 42 variables) had *P*-values ≤0.05 (Supplementary Table 1, see Additional file 1). After correction of *P*-values for multiple testing, 57 of these associations remained significant (Table 2), which did not include any associations with arginine. Thirty associations were replicated in the validation set (same direction as in the discovery set, Supplementary Table 1, see Additional file 1) after Bonferroni correction of *P*-values, which did not include any associations with PC aa C36:3 (Table 2).

**Table 2:** *P*-values for associations between metabolites and selected^a^ variables

Metabolite	Variable	Discovery		Validation	
		*P*-value	minP *P-*value	*P*-value	Bonf. *P*-value
Arginine^b^	*-*	**-**	**-**	**-**	**-**
Asparagine	**BMI**	**1.4E−19**	**1.4E−19**	**9.3E−07**	**5.6E−06**
	**Waist circumference**	**4.8E−16**	**4.8E−16**	**9.9E−05**	**6.0E−04**
	**Hip circumference**	**2.2E−13**	**2.2E−13**	**3.7E−03**	**2.2E−02**
	**Waist/hip ratio**	**1.3E−05**	**1.0E−03**	**8.2E−03**	**4.9E−02**
	Fiber intake	1.3E−03	3.9E−02	1.5E−01	8.9E−01
	WCRF/AICR score	8.3E−04	2.4E−02	5.0E−01	1.0E+00
C2	**Age at blood collection**	**8.6E−04**	**2.6E−02**	**2.3E−04**	**1.1E−03**
	Alcohol consumption at recruitment	2.8E−04	8.0E−03	2.0E−01	1.0E+00
	Alcohol consumption (lifetime)	8.6E−04	2.6E−02	3.5E−01	1.0E+00
	Carbohydrate intake	3.8E−05	1.0E−03	3.0E−01	1.0E+00
	Total sugar intake	2.1E−04	6.0E−03	1.1E−01	5.5E−01
PC aa C36:3	Age at blood collection	1.0E−03	4.3E−02	1.2E−01	2.4E−01
	BMI	8.9E−04	3.6E−02	6.7E−02	1.3E−01
PC ae C34:2	Smoking status	1.2E−04	1.0E−03	7.5E−01	1.0E+00
	Smoking status and intensity	2.0E−04	3.0E−03	8.4E−01	1.0E+00
	**BMI**	**5.7E−10**	**45.7E−10**	**6.6E−05**	**7.9E−04**
	**Waist circumference**	**2.6E−14**	**2.6E−14**	**3.0E−07**	**3.6E−06**
	Hip circumference	4.3E−05	4.3E−05	2.2E−02	2.6E−01
	**Waist/hip ratio**	**5.0E−12**	**5.0E−12**	**3.3E−07**	**3.9E−06**
	Carbohydrate intake	4.3E−06	4.3E−06	4.2E−02	5.0E−01
	Total sugar intake	1.4E−03	3.2E−02	9.8E−01	1.0E+00
	**Total fat intake**	**2.2E−09**	**2.2E−09**	**1.4E−04**	**1.7E−03**
	**Fatty acids, total saturated intake**	**6.8E−10**	**6.8E−10**	**8.7E−07**	**1.0E−05**
	Fatty acids, total monounsaturated intake	1.5E−04	1.0E−03	1.2E−01	1.0E+00
	Modified Mediterranean diet score	5.1E−04	1.5E−02	9.5E−03	1.1E−01
PC ae C36:2	Smoking status	2.1E−04	6.0E−03	6.1E−01	1.0E+00
	Smoking status and intensity	2.4E−04	8.0E−03	9.0E−01	1.0E+00
	**Alcohol consumption at recruitment**	**4.4E−08**	**4.4E−08**	**1.4E−04**	**1.7E−03**
	**Alcohol consumption (lifetime)**	**1.3E−06**	**1.3E−06**	**6.8E−04**	**8.1E−03**
	**BMI**	**4.4E−13**	**4.4E−13**	**7.0E−09**	**8.4E−08**
	**Waist circumference**	**2.1E−19**	**2.1E−19**	**1.8E−12**	**2.2E−11**
	**Hip circumference**	**1.1E−07**	**1.1E−07**	**7.7E−05**	**9.3E−04**
	**Waist/hip ratio**	**9.1E−15**	**9.1E−15**	**7.0E−10**	**8.4E−09**
	**Total fat intake**	**1.4E−11**	**1.4E−11**	**2.1E−05**	**2.5E−04**
	**Fatty acids, total saturated intake**	**2.2E−17**	**2.2E−17**	**1.6E−10**	**1.9E−09**
	**Healthy Lifestyle Index**	**2.8E−05**	**2.0E−03**	**2.3E−04**	**2.8E−03**
	**WCRF/AICR score**	**3.9E−05**	**2.0E−03**	**8.1E−05**	**9.7E−04**
PC ae C36:3	Smoking status	3.8E−04	7.0E−03	8.1E−01	1.0E+00
	Smoking status and intensity	1.4E−03	2.8E−02	8.6E−01	1.0E+00
	**BMI**	**1.9E−07**	**1.9E−07**	**2.2E−03**	**2.7E−02**
	**Waist circumference**	**7.2E−12**	**7.2E−12**	**7.3E−05**	**8.8E−04**
	Hip circumference	4.7E−04	8.0E−03	6.8E−02	8.1E−01
	**Waist/hip ratio**	**1.5E−10**	**1.5E−10**	**8.2E−05**	**9.8E−04**
	Carbohydrate intake	1.9E−04	5.0E−03	2.2E−01	1.0E+00
	**Total fat intake**	**8.5E−08**	**8.5E−08**	**4.1E−03**	**4.9E−02**
	**Fatty acids, total saturated intake**	**1.5E−06**	**1.5E−06**	**1.6E−03**	**2.0E−02**
	Fatty acids, total monounsaturated intake	2.8E−05	2.8E−05	5.0E−02	6.0E−01
	Fatty acids, total trans intake	1.4E−04	3.0E−03	2.1E−01	1.0E+00
	Modified Mediterranean diet score	3.0E−04	6.0E−03	5.6E−02	6.7E−01
PC ae C38:2	Alcohol consumption at recruitment	3.4E−04	2.4E−02	1.2E−02	9.9E−02
	**BMI**	**6.2E−11**	**6.2E−11**	**1.9E−09**	**1.5E−08**
	**Waist circumference**	**1.6E−13**	**1.6E−13**	**2.5E−11**	**2.0E−10**
	**Hip circumference**	**7.6E−08**	**7.6E−08**	**1.2E−06**	**9.6E−06**
	**Waist/hip ratio**	**1.1E−07**	**1.1E−07**	**3.6E−06**	**2.9E−05**
	Total fat intake	2.5E−04	1.7E−02	9.2E−03	7.3E−02
	**Fatty acids, total saturated intake**	**1.7E−06**	**1.7E−06**	**1.2E−04**	**1.0E−03**
	Fatty acids, total trans-polyenoic intake	1.2E−03	4.9E−02	6.3E−01	1.0E+00

Bold lines correspond to variable showing significant association after adjustment of *P*-value for multiple tests in both discovery and validation sets. Models were adjusted for center of recruitment, age, menopausal status (premenopausal, perimenopausal, postmenopausal), phase of the menstrual cycle for premenopausal women (follicular, ovulatory, luteal, missing), time of the day, and fasting status at blood collection (no, in between, yes)

*Abbreviations*: *AICR* American Institute for Cancer Research, *BMI* body mass index, *C2* acetylcarnitine, *PC aa* phosphatidylcholine diacyl, *PC ae* phosphatidylcholine acyl-alkyl, *WCRF* World Cancer Research Fund

^a^Only associations for which a significant *P*-value was detected after correction of *P*-values for multiple tests in the discovery sets are included

^b^No association was detected in the discovery set

**Fig. 2:**
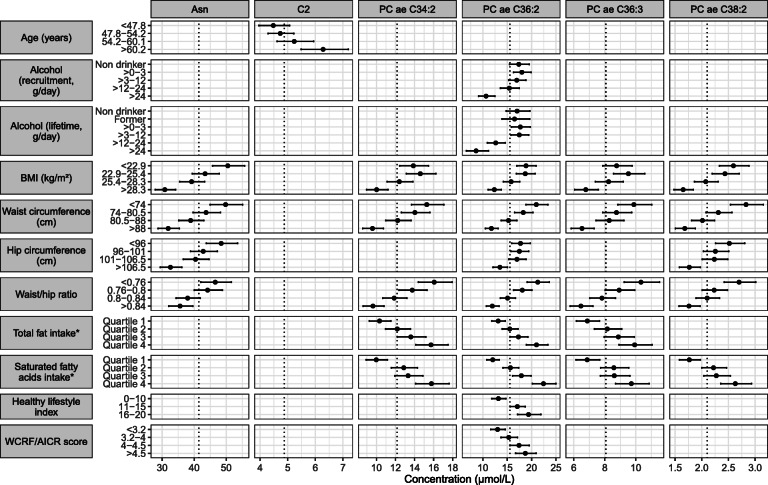
Adjusted means of metabolite concentrations by categories of correlates (*N* = 2358). Only metabolites and variables for which a significant association was detected in the discovery and validation sets are shown. Adjusted means and their 95% confidence intervals were obtained from linear regression models adjusted for fasting status, center, age, date and time at blood collection, menopausal status, and phase of menstrual cycle at blood collection. Dotted lines indicate the overall means of metabolite concentration. *Residuals on total energy intake. Abbreviations: Asn, asparagine; AICR, American Institute for Cancer Research; BMI, body mass index; C2, acetylcarnitine; PC aa, phosphatidylcholine diacyl; PC ae, phosphatidylcholine acyl-alkyl; WCRF, World Cancer Research Fund

Figure 2 represents means of the metabolite concentrations across categories of variables in the overall population (*n* = 2358), for metabolites and variables for which a significant association was detected in both the discovery and validation sets. Asparagine concentration was negatively associated with BMI, waist and hip circumferences, and WHR. C2 was positively associated with age but not with the other factors. PCs ae C36:2 and ae C38:2 were negatively associated with BMI, waist and hip circumferences, and waist/hip ratio. Negative associations with BMI, waist circumference, and waist/hip ratio were also observed for PCs ae C34:2 and ae C36:3. PC ae C34:2, C36:2, and 36:3 were additionally positively associated with total fat intake, and with saturated fatty acid intake, which was also positively associated with PC ae C38:2. For PC ae C36:2, additional associations were observed with alcohol intake at recruitment and over lifetime (negative) and with HLI and WCRF/AICR score (positive).

Analyses of interactions with BMI, menopausal, and fasting status (Supplementary Table 2, see Additional file 1) did not suggest any significant interaction with these variables in the associations reported above. The only interactions with significant *P*-values after correction for multiple testing were with menopausal status for the association between asparagine and age (P-int = 0.04) and with fasting status for the association of height and PC ae C38:2 (P-int = 0.03).

When excluding serum samples (restricting the analysis to plasma samples) from both discovery (*n* = 40) and validation (*n* = 88) sets, results were largely consistent with those in the main analyses (data not shown), except for generally larger *P*-values (due to the lower statistical power) that led to the following non-significant associations in the discovery set: asparagine and WCRF/AICR score, PC aa C36:3 and age and BMI, and PC ae C38:2 and trans-polyenoic fatty acid intake. In the validation set, associations between asparagine and hip and waist circumferences were not statistically significant anymore. However, estimates were very close in direction and magnitude to the ones obtained overall (before exclusion of serum samples).

After exclusion of participants with self-reported diabetes at blood collection (discovery set, *n* = 45; validation set, *n* = 26) or with missing information on diabetes (discovery set, *n* = 86; validation set, *n* = 74), associations were very similar in direction and magnitude to those observed in the whole dataset, although sometimes not significant in the validation set (data not shown), such as asparagine and hip circumference and waist/hip ratio, and PC ae C36:3 and BMI and total and saturated fat intakes.

## Discussion

In this study, we identified several lifestyle and anthropometric correlates of blood metabolites which have been previously associated with breast cancer risk in women not taking exogenous hormones at blood collection. Concentrations of PCs ae C34:2, ae C36:2, ae C36:3, and ae C38:2 showed negative associations with adiposity and positive associations with total (except for PC ae C38:2) and saturated fat intakes. PC ae C36:2 also showed a negative association with alcohol consumption and positive associations with the WCRF/AICR score and the Healthy Lifestyle Index. Asparagine concentrations were negatively associated with adiposity, and arginine concentrations were not associated with any of the variables examined. Acetylcarnitine concentrations were positively associated with age but not with any of the other factors. We did not identify any correlate of the only diacyl PC (PC aa C36:3) associated with breast cancer risk. These associations were consistent across different BMI, fasting status, and menopausal status categories.

Acyl-alkyl phosphatidylcholines have been previously associated with various lifestyle and dietary factors. In our work, concentrations of acyl-alkyl PCs were negatively associated with measures of adiposity (including BMI and waist circumference). This observation is consistent with the global pattern of negative associations between PCs ae and BMI previously reported in EPIC [12], in particular for PCs ae C38:2 [29] and ae C36:2 [30], and in the EPIC-Potsdam sub-cohort [31]. PC ae C38:2 and C34:2 were also associated with weight loss in an intervention study (*n* = 17 participants) [12]. In the EPIC-Potsdam sub-cohort [32], a negative association of several PCs ae was reported with risk of type 2 diabetes, as well as a positive correlation with circulating high-density lipoprotein cholesterol. In an analysis of two studies of Japanese and American men and women [33], PCs ae C34:2, 36:3, and 38:2 were negatively associated with metabolic syndrome (in particular with high-density lipoprotein cholesterol and triglycerides), but not with elevated waist circumference. Among 200 Canadian adults younger than 55 years, concentrations of PCs ae C34:2, C36:2, and C36:3 were lower in obese participants with metabolic syndrome than in obese participants without metabolic syndrome and in normal weight participants [34], while an opposite trend was reported for several PCs aa. These results support an association of PCs with obesity or metabolic health that deserves further investigation.

Lower concentrations of PCs were reported in vegetarian and vegan men than in meat eaters [35]. Moreover, analyses in colorectal cancer patients (60% males) indicated positive associations of several PCs, mostly acyl-alkyl, with Western and carnivore dietary patterns [36]. These results are consistent with the positive association we report with saturated fat intake. However, few studies have been conducted in women, and an analysis conducted among healthy participants from the KarMeN study, not using exogenous hormones, suggested differences in plasma concentrations of some PCs between men and women, although PCs were not the most important components for predicting sex [37]. A recent metabolomic study of plasma lipid-related profiles and diet quality in the Nurses’ Health Study [38] reported that PC C36:2 plasmalogen was associated with unhealthy components of the Alternate Healthy Eating Index.

A negative association of PCs and alcohol consumption, in particular PC ae C36:2, has been reported in EPIC, in both men and women [39]. A negative association with PC ae C36:2 was also observed separately in men and women from the KORA F4 study when comparing moderate-to-heavy drinkers (≥20 g/day for women, 40 g/day in men) with light drinkers (< 20 g/day for women, 40 g/day in men) [40], and in the CARLA study (men and women combined) [41].

The positive associations reported between PC ae C36:2 and the WCRF/AICR and HLI scores, which integrate alcohol and body weight components, likely reflect inverse associations of this metabolite with alcohol consumption and adiposity as demonstrated in the analyses of single correlates. These associations are in line with a recent study conducted in EPIC on metabolic signatures of a healthy lifestyle, assessed by the WCRF/AICR score [42]. In this work, PCs ae 36:2 and C38:2 were among endogenous metabolites with the greatest loadings (> 100 examined) in the signature of the WCRF/AICR score. This metabolic signature showed the greatest correlations with the recommendations regarding normal weight maintenance and alcohol avoidance, in line with the associations we report. In contrast, a study in colorectal cancer patients indicated negative associations between several PCs ae and aa and the WCRF/AICR score [36]. However, the score was restricted to its dietary components, therefore not considering the body weight component.

Metabolomics studies on aging reported increasing circulating concentrations of acylcarnitines, mostly long-chain, with age [43, 44], which could reflect loss in mitochondrial function [45]. In a study [46] comparing metabolites in serum samples obtained 7 years apart from the same individuals (KORA S4 and KORA F4), acetylcarnitine and several other acylcarnitines increased in the follow-up samples compared with baseline samples. Associations of similar direction were observed in their validation study on samples collected 4 years apart, although not statistically significant after accounting for multiple testing. Acylcarnitines have also been associated with impaired glucose metabolism and insulin resistance, but these associations were most often reported for long-chain or odd short-chain acylcarnitines [47–51], although associations with acetylcarnitine (which is an even short-chain acylcarnitine) have also been reported [52]. In our previous work, this metabolite was the only one to show a positive association with breast cancer risk in age-matched cases and controls, suggesting that its association with age does not fully explain the association with breast cancer. In the present work, we did not observe any association of acetylcarnitine with anthropometric factors likely associated with metabolic health, in contrast with a positive association with BMI reported in the EPIC Norfolk cohort [53].

A negative association between circulating asparagine and obesity has been recently reported in different populations, including Europeans [50, 53], obese Iranian adults [54], and Japanese [55]. Negative associations with diabetes and coronary artery disease have also been reported [50, 53], in lean as well as in obese subjects [49]. However, most studies exploring the associations between amino acids and obesity showed significant associations only with branched-chain amino acids (which do not include asparagine) [49, 56]. Asparagine was also part of the metabolic signature of a healthy lifestyle derived in EPIC [42] and of the metabolic signature of BMI, waist circumference, and waist/hip ratio [12].

In our study, arginine was not associated with any of the factors investigated. This result contrasts with those in several studies reporting negative associations of arginine with age [46] and with obesity and alcohol intake, as well as a positive association with smoking in the EPIC Norfolk cohort [53], which however had not excluded hormone users. Arginine has also been negatively associated with hemoglobin concentrations and with insulin-like growth factor 1 and estradiol [57] in premenopausal women not using exogenous hormones. These observations may suggest that arginine concentrations could potentially be more tightly regulated by endogenous metabolism compared to lifestyle exposures.

Major strengths of this work include the wide variety of data collected which enabled us to investigate many potential correlates for the metabolites associated with breast cancer risk, and the large sample size of our study, compared to other metabolomics studies, where large studies are essential [58]. With the detailed information available on characteristics of women at blood collection, we were also able to exclude hormone users from our analysis, which is important as hormone use could possibly affect concentrations of some metabolites [59].

A first limitation to this work is the cross-sectional design, which prevents us from drawing any conclusions on the timing or causality of the associations. Another limitation is that the large sample size was achieved by pooling data from different previous studies, rather than by initial design, therefore adding methodological complexity because of analyses performed by different laboratories, with different instruments, and on different biological matrices. However, the analytical protocol used has shown high inter-laboratory reproducibility [60], and we addressed potential heterogeneity in metabolite concentrations by developing a dedicated pipeline [24] applied to the data prior to statistical analyses. In addition, for all metabolites included (except asparagine, not evaluated), high correlations were reported between measures in serum and in plasma (*r* ≥ 0.78, except for arginine, *r* = 0.50), although concentrations were generally higher in serum than in plasma, in particular for arginine [61]. Good reliability of measurements was also reported for both matrices (intra-class correlations for the metabolites of interest ≥0.58 in plasma, ≥0.67 in serum) [62]. Furthermore, exclusion of serum samples did not substantially modify the results. A third limitation is the heterogeneity of fasting status of participants. However, variables to determine fasting status were carefully recorded, therefore enabling us to test the effect of this variable on the results, and we found no evidence of heterogeneity in the associations by fasting status. Dietary intakes were assessed using food frequency questionnaires adapted to local habits. These questionnaires were validated through a calibration approach using a common 24-h diet recall [63] to adjust for possible systematic misclassification in dietary measurements, and a validation study using 24-h urine samples was conducted [64]. Despite these methodological efforts, however, potential measurement error may persist because of recall bias, misreporting of consumption for certain foods, or errors related to the food composition tables used (despite careful matching [15]). Nevertheless, several cross-sectional studies showing good correlations [65, 66] between intakes measured by food questionnaires and expected specific biomarkers suggest that data from food frequency questionnaires can be used for the purposes of the present work. Finally, the applied technology for PC measurement does not allow for precise identification of the compounds measured, since the signal observed is not specific and may correspond to different structural isomers. Further work is needed to investigate specifically associations with lipid compounds.

## Conclusions

In conclusion, this cross-sectional analysis identified several modifiable correlates of blood concentrations of metabolites associated with breast cancer risk. These associations may indicate possible mechanisms underlying associations between lifestyle and anthropometric factors, and risk of breast cancer. To better understand how our results could improve our current knowledge on the association between lifestyle factors and breast cancer risk, dedicated tools, such as mediation analysis, bring promising perspectives. Intervention studies would be required to evaluate the possible causality of the associations observed with modifiable factors and to assess whether concentrations of these specific metabolites could be modified through lifestyle changes.

## Supplementary Information

**Additional file 1:** Associations between metabolites and all variables tested as correlates, in discovery set and, for significant associations, in validation set (Supplementary Table 1); Stratified analyses by BMI, menopausal status, and fasting status at blood collection, for metabolites and variables showing a P-int < 0.20 after correction for multiple testing, in discovery set (Supplementary Table 2). Abbreviations: AICR American Institute for Cancer Research; Asn asparagine; BMI body mass index; C2 acetylcarnitine; HLI Healthy lifestyle index; PC aa phosphatidylcholine diacyl; PC ae phosphatidylcholine acyl-alkyl; SD Standard deviation; SE: Standard error of estimate; WCRF World Cancer Research Fund. (XLS 177 kb)

## Data Availability

EPIC data are available for investigators who seek to answer important questions on health and disease in the context of research projects that are consistent with the legal and ethical standard practices of IARC/WHO and the EPIC Centres. The primary responsibility for accessing the data belongs to the EPIC centers that provided them. For information on how to submit an application for gaining access to EPIC data and/or biospecimens, please follow the instructions at http://epic.iarc.fr/access/index.php.

## Abbreviations

AICR: American Institute for Cancer Research
Asn: Asparagine
BMI: Body mass index
C2: Acetylcarnitine
*CI*: Confidence interval
EPIC: European Prospective Investigation into Cancer and Nutrition
HLI: Healthy Lifestyle Index
IARC: International Agency for Research on Cancer
LLOQ: Lower limit of quantification
PC aa: Phosphatidylcholine diacyl
PC ae: Phosphatidylcholine acyl-alkyl
SD: Standard deviation
WCRF: World Cancer Research Fund
